# Antibacterial effects of bio-inspired nanostructured materials

**DOI:** 10.1080/20002297.2017.1325241

**Published:** 2017-06-09

**Authors:** Monika Astasov-Frauenhoffer, Khaled Mukaddam, Irmgard Hauser-Gerspach, Joachim Köser, Thilo Glatzel, Marcin Kisiel, Laurent Marot, Sebastian Kühl

**Affiliations:** ^a^ Department of Preventive Dentistry and Oral Microbiology, University Center for Dental Medicine, University of Basel, Basel, Switzerland; ^b^ Department of Oral Surgery, Oral Radiology and Oral Medicine, University Center for Dental Medicine, University of Basel, Basel, Switzerland; ^c^ Institut für Chemie und Bioanalytik, Hochschule für Life Sciences, Muttenz, Switzerland; ^d^ Department of Physics, University of Basel, Basel, Switzerland

## Abstract

Several properties of bio-inspired surfaces like chemical composition, surface topography, surface hydrophilicity and even surface charge could influence bacterial adhesion to implant materials. Therefore, a nanostructured surface is being investigated to avoid bacterial colonization by their physico-mechanical and chemical aspects.

Both smooth and rough-surfaced titanium (PT, SLA) and zirconia (M and ZLA) surfaces were used as controls. Titanium SLA was modified by two-step-etching to create nanostructured surface. Antibacterial properties of the materials were tested by adhesion of *Porphyromonas gingivalis* (ATCC 33277). The vitality of bacteria was assessed by Live/Dead BacLight™ Bacterial Viability Kit or by conventional culturing on Columbia blood agar.

Conventional culturing revealed reduction of bacteria on nanostructured titanium (5.27±0.8 x 10^4^ CFU/mm^2^) in comparison to rough-surfaced control materials (ZLA 6.16±4.86 x 10^4^ and SLA 1.53±0.75 x 10^5^ CFU/mm^2^). However, smooth-surfaced control materials (M 2.25±0.84 x 10^4^ and PT 6.63±5.77 x 10^3^ CFU/mm^2^) showed similar results to the nanostructured material. Live/dead staining demonstrated the antimicrobial efficacy of the nanostructured material revealing reduction of vital bacteria population up to 70%. This effect was not observed on the control materials (bacterial vitality ≥95%).

In conclusion, nanostructured titanium surface shows a reduction of vital bacteria. Therefore, bio-inspired nanostructures can modify the bacteria–titanium interaction.

